# Octreotide Uptake in Parathyroid Adenoma

**DOI:** 10.4274/Mirt.4

**Published:** 2012-08-01

**Authors:** Seyhan Karaçavuş, Mustafa Kula, Züleyha Cihan Karaca, Kürşad Ünlühızarcı, Ahmet Tutuş, Fahri Bayram, Ganime Çoban

**Affiliations:** 1 Bozok University School of Medicine, Nuclear Medicine Department of, Yozgat, Turkey; 2 Erciyes University School of Medicine, Endocrinology Department of, Kayseri, Turkey; 3 Erciyes University Erciyes School of Medicine, Pathology Department of, Kayseri, Turkey

**Keywords:** Parathyroid adenoma, indium-111-octreotide, somatostatin

## Abstract

The patient with a history of bone pain and muscle weakness, was thought to have oncogenic osteomalacia as a result of biochemical investigations and directed to Nuclear Medicine Department for a whole-body bone scintigraphy and 111In-octreotide scintigraphy. There was no focal pathologic tracer uptake, but generalized marked increase in skeletal uptake on bone scintigraphy. Octreotide scintigraphy showed accumulation of octreotide in the region of the left lobe of the thyroid gland in the neck. Thereafter, parathyroid scintigraphy was performed with technetium-99m labeled metroxy-isobutyl-isonitryl (99mTc-MIB) and MIBI scan demonstrated radiotracer uptake at the same location with octreotide scintigraphy. The patient underwent left inferior parathyroidectomy and histopathology confirmed a parathyroid adenoma. Somatostatin receptor positive parathyroid adenoma may show octreotide uptake. Octreotide scintigraphy may be promising and indicate a possibility of using somatostatin analogues for the medical treatment of somatostatin receptor positive

**Conflict of interest:**None declared.

## INTRODUCTION

111In-octreotide scintigraphy, based on the expression of somatostatin receptors by the tumor cells, is generally used to detect neuroendocrine tumors (1). Many other situations like primary or metastatic thyroid cancers, Hashimato thyroiditis, endemic goiters and normally functioning thyroid nodules were described to show octreotide uptake (2,3). However, octreotide scintigraphy rarely demonstrates an accumulation of radiotracer on parathyroid gland. Somatostatin immunoreactivity has been described in human parathyroid gland but whether somatostatin receptors are present or not is still unclear (4,5). In previous studies, autoradiography revealed specific expression of somatostatin receptor binding of radiolabeled octreotide in peritumoral veins of parathyroid tumors but not in parathyroid tumor cells (6). Afterwards, Faggiano et al reported in their study which investigated the effectiveness of therapy with somatostatin analogues on primary hyperparathyroidism, that octreotide scintigraphy showed positive parathyroid tumor uptake in three of eight patients with Multiple Endocrine Neoplasia (MEN) 1 syndrome (7). We describe a case of a patient with parathyroid adenoma demonstrated on 111In-octreotide scintigraphy.

## CASE REPORT

A 48-year-old woman was presented to our department with history of bone pain, muscle weakness. Biochemical investigations showed hypophosphatemia (1.8 mg/dl, range 2.5-4.8); increased alkaline phosphatase (1059 U/l, range 38-126); normocalcaemia (10.2 mg/dl, range 8.9-10.3) supporting hypocalcaemic onkogenic osteomalacia.

**Figure 1:**
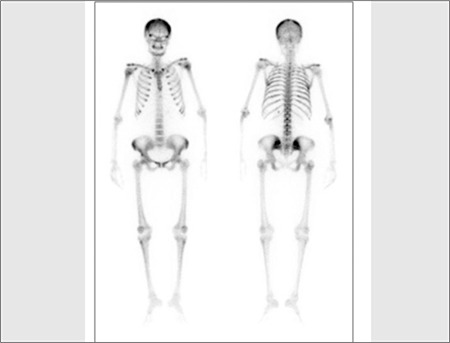
The generalized marked in skeletal uptake on anterior andposterior view of the whole-body bone scan (superscan appearance)

**Figure 2:**
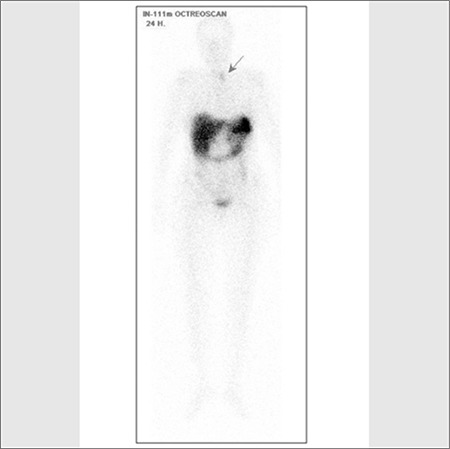
^111^In octreotide whole body scintigraphy revealed focaluptake at the localization of the left thyroid lobe in the neck

**Figure 3:**
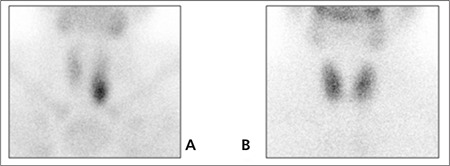
^99m^Tc-MIBI (A)/^99^Tc pertechhnetate (B) prathyroid imagingdemons intense MIBI retention in left lobe inferior of thyroid glandsupporting parathyroid aden

**Figure 4:**
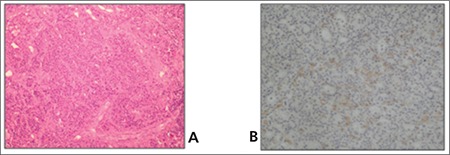
Parathyroid adenoma with formation of microfollicles(H&Ex 200) (A) and Somatostatin Ab-1 immunreactivity positive areasin parathyroid adenoma (IHCx400) (B)

Whole body bone scintigraphy was performed after intravenous administration of 20 mCi (740 MBq) of methyleno-diphosphonate labeled with technetium-99m (Tc- 99m MDP). Bone scan showed metabolic bone disorder featured by the following characteristics: Prominent tracer uptake in the calvarium, mandible, spine, rib, and bilateral femora; intense uptake at the costochondral junctures and faint kidney visualization (Figure 1). Besides, whole body scintigraphy was performed up to 48 hours after the intravenous administration of 5 mCi (185 MBq) 111In-octreotide. Octreotide scan showed accumulation of octreotide in the region of the left lobe of the thyroid gland, but there was no other pathologic tracer accumulation (Figure 2). Then, a neck ultrasonography was performed in which a well-defined nodular lesion was seen outside of the left thyroid gland and there were no thyroid nodules. We thought it could be a parathyroid adenoma. PTH level was 1686.8 pg/ml. Then, we decided to perform a parathyroid scintigraphy with 99mTc MIBI. MIBI scan demonstrated radiotracer uptake at the same location with octreotide scan in the neck (Figure 3 A, B). For that reason, parathyroidectomy was performed. A parathyroid adenoma was confirmed histopatologically. Pathologic examination of the lesion showed immunoreactivity with Somatostatin Ab-1 (Figure 4 A, B). PTH level decreased to 82.5 pg/ml after surgery. 

## LITERATURE REVIEW AND DISCUSSION

Parathyroid adenoma is part of a spectrum of parathyroid proliferative disorder that includes parathyroid hyperplasia, parathyroid adenoma and parathyroid carcinoma (8). Eighty to 85 percent of primary hyperparathyroidism is caused by parathyroid adenoma followed by primary parathyroid hyperplasia (15%) and parathyroid carcinoma (5%) (8).

Patients with primary hyperparathyroidism may present clinical evidence of elevated serum calcium levels which include non-specific symptoms such as fatigue, pain and weakness as well as polydipsia, polyuria, and nephrolithiasis (9). Sonography and dual-phase 99mTc-sestamibi scintigraphy are the primary imaging modalities preoperatively utilized for the visualization of diseased glands (10).However, as in the case presented here, somatostatin immunoreactivity was rarely described in human parathyroid adenomas (4). Some tumor tissues including endocrine tumors express somatostatin receptors, and this provides the basis of the utilization of somatostatin analogues for detecting and treatment of these tumors as well as their metastasis by means of scintigraphy (8).

Some studies also have suggested a role for somatostatin analogues in the medical treatment of hyperparathyroidism (7,12,13). Faggiano et al. reported in their study which investigated the effectiveness of therapy with somatostatin analogues on primary hyperparathyroidism, that octreotide scintigraphy showed positive parathyroid tumor uptake in three of eight patients with MEN 1 syndrome and six months of depot long-acting octreotide (OCT-LAR) therapy controlled hypocalcaemia and hypercalciuria in two-thirds of patients with MEN 1-related primary hyperparathyroidism (7). Asnacios et al. revealed that a positive correlation has been found between tumor expression of somatostatin receptor-2 and positive results (positive fixation of primary and metastasis) at scintigraphy using radiolabeled octreotide (14). Conversely, Zielke et al. reported that octreotide has no effect on biochemical parameters in patients with hyperparathyroidism (5). They maintained that somatostatin receptors are absent, together with lack of octreotide effects and somatostatin analogues are not effective in the medical therapy of hyperparathyroidism. In our case, somatostatin immunoreactivity was shown by pathologic examination in parathyroid gland and octreotide scan was positive.

In conclusion, when a focal uptake was observed in the region of the thyroid gland on octreotide scintigraphy, the presence of parathyroid adenoma should also be considered. Parathyroid tumors expressing somatostatin receptors may show octreotide uptake and octreotide scan may be promising and indicate a possibility of using somatostatin analogues for medical treatment of somatostatin receptor positive parathyroid tumors in some situations which surgery is not possible and parathyroid tumor recurrence.
